# The impact of chatbots based on large language models on second language vocabulary acquisition

**DOI:** 10.1016/j.heliyon.2024.e25370

**Published:** 2024-02-01

**Authors:** Zhihui Zhang, Xiaomeng Huang

**Affiliations:** a Rossier School of Education, University of Southern California, 3551 Trousdale Pkwy, Los Angeles, CA, 90089, USA; b Alibaba Cloud, 969 West Wen Yi Road Yu Hang District, Hangzhou, Zhejiang Province, 311121, China

**Keywords:** Large language models, Chatbot, Second language acquisition, Vocabulary learning

## Abstract

In recent years, the integration of artificial intelligence (AI) and machine learning (ML) into education, particularly for Personalized Language Learning (PLL), has garnered significant attention. This approach tailors interventions to address the unique challenges faced by individual learners. Large Language Models (LLMs), including Chatbots, have demonstrated a substantial potential in automating and enhancing educational tasks, effectively capturing the complexity and diversity of human language. In this study, 52 foreign language students were randomly divided into two groups: one with the assistance of a Chatbot based on LLMs and one without. Both groups learned the same series of target words over eight weeks. Post-treatment assessments, including systematic observation and quantitative tests assessing both receptive and productive vocabulary knowledge, were conducted immediately after the study and again two weeks later. The findings demonstrate that employing an AI Chatbot based on LLMs significantly aids students in acquiring both receptive and productive vocabulary knowledge during their second language learning journey. Notably, Chatbots contribute to the long-term retention of productive vocabulary and facilitate incidental vocabulary learning. This study offers valuable insights into the practical benefits of LLM-based tools in language learning, with a specific emphasis on vocabulary development. Chatbots utilizing LLMs emerge as effective language learning aids. It emphasizes the importance of educators understanding the potential of these technologies in L2 vocabulary instruction and encourages the adoption of strategic teaching methods incorporating such tools.

## 1 Introduction

In recent years, the integration of artificial intelligence (AI) in education has gained a substantial attention, and emerged as a powerful tool in education, addressing the myriad challenges faced by educators and students while enhancing the quality of the learning experience [1]. Prominent trends in this domain include the digitization of educational materials, gamification, and tailored learning experiences, demonstrating the extensive opportunities for AI applications in the field of education.

Within the realm of AI applications in education, a notable subfield is AI Chatbots. These intelligent systems, capable of performing a wide range of tasks, have attracted significant interest in recent years. Chatbots, often referred to as smart bots, interactive agents, digital assistants, or artificial conversation entities, utilize AI to mimic human conversation [2]. Researchers have examined their applications, design guidelines, evaluation possibilities, and the effects in education. It is evident that AI Chatbots offer immediate, real-time interactions, thereby enhancing the overall learning experience for users [3,4].

Although there is an enthusiasm for the potential of AI chat robots in education, it's essential to recognize the existing limitations. Recent studies have demonstrated the potential transformation of chatbots into intelligent teaching assistants [5]. However, previous AI Chatbots often struggle to comprehend natural language without specific commands or keyword searches. Furthermore, they encounter challenges in generating varied responses tailored to different student queries [6,7]. Moreover, the concept of chatbots as language learning tools is still not widely known, with research primarily focusing on learning outcomes and performance improvement. Studies, especially empirical research, examining their effectiveness as language learning tools, particularly for second-language learners, are scarce. This limitation can be attributed to the constraints of previous machine learning technologies, which struggled with personalization and the consideration of language proficiency levels.

This study aims to address these research gaps by utilizing Chatbots Based on Large Language Models (LLMs) on language. Unlike traditional machine learning systems, Chatbots based on LLMs can mimic the complexity of human language and provide real-time responses. Recent studies have shown their versatility in various domains, including education [8,9]. Additionally, Chatbots (LLMs) have undergone optimization to reduce the generation of irrelevant information and outdated knowledge after training [10]. They have the potential to offer personalized language assistance, thus bridging the gaps in interactive teaching and learning support.

The importance and novelty of this study lie in its investigation of the impact of Chatbots (LLMs) on second-language learners, with a focus on vocabulary. This study assesses various aspects of language learning, including L2 proficiency (receptive and productive vocabulary knowledge), and learning processing (incidental learning). 10.13039/100014337Furthermore, the role of AI as a teaching assistant in supporting students' self-regulated learning, a largely unexplored area, is explored. Self-regulation challenges, a significant concern in language learning environments, can potentially be mitigated by AI support [11]. Hence, this study also analyzes the effectiveness of Chatbots (LLMs) in facilitating second-language incidental learning. It aims to provide valuable insights into the practical application and benefits of these Chatbots in language learning.

The research questions are listed below. By addressing these questions, this study aims to bridge essential gaps in the existing research. It aims to offer valuable insights into the efficacy and potential advantages of Chatbots (LLMs) in improving language proficiency and facilitating self-regulated learning among students. In terms of research on whether Chatbots can enhance productive vocabulary, this study can push the boundaries of chatbot roles in teaching beyond being just assessment tools.

RQ1 To what extent do Chatbots (LLMs) enhance receptive vocabulary knowledge in second language (L2) learners?

RQ2 To what extent do Chatbots (LLMs) enhance productive vocabulary knowledge in L2 learners?

RQ3 How do Chatbots (LLMs) contribute to the promotion of incidental vocabulary learning in L2 learners?

The research work consists of six sections. Section 1 is the introduction, which elaborates on the research background, research methods and processes, research objectives, research innovation, and research framework of chat robots in education. Section 2 is a literature review, which mainly reviews receptive and productive vocabulary knowledge, incidental vocabulary learning, AI chatbots in language learning, LLM, and Reinforcement Learning from Human Feedback (RLHF). It indicates that this study aims to optimize LLM to provide insights for personalized language education. Section 3 is the research methodology section, which mainly adopts a mixed approach, combining qualitative and quantitative methods, to evaluate the impact of Chatbots (LLMs) on vocabulary acquisition of English learners. Section 4 is the research results section, which mainly visualizes and analyzes the experimental results. Section 5 is the finding and discussion section, which mainly combines relevant research to conduct in-depth discussions on the research results. Section 6 is the conclusion section, including research results, research significance, research shortcomings, and future prospects.

## 2 Literature review

### 2.1 Receptive and productive vocabulary knowledge

In the realm of language acquisition, vocabulary acquisition holds a pivotal role, whether in one's native language or a second language (L2). The process of acquiring a second language significantly relies on vocabulary, as it forms the fundamental building blocks from which learners embark on their journey towards mastering the L2.

In language learning, Nation distinguishes between receptive vocabulary and productive vocabulary. Receptive vocabulary refers to words that learners can understand and recognize, though they may not necessarily actively use or express. Productive vocabulary comprises words that learners can actively use and apply in spoken or written language, whether in conversation or writing [12]. For instance, if a learner can comprehend and translate the English word “computer” in their first language but struggles to independently express “computer” in English, it falls under receptive vocabulary. Conversely, if a learner can articulate and use “computer” in English, it becomes part of their productive vocabulary. Typically, learners possess a more extensive receptive vocabulary than productive vocabulary, primarily because they frequently encounter new vocabulary during reading and listening comprehension, even if they do not necessarily employ them in language output [13]. Thus, the receptive-productive dimension reflects the learner's control and access to knowledge about words.

Enhancing productive vocabulary, especially among second language learners, has been a longstanding research focus. Traditional studies have primarily concentrated on the relationship between teaching methods and receptive vocabulary size. However, research on improving productive vocabulary, especially through Computer-Assisted Language Learning (CALL) [14], remains relatively limited. Prior research on CALL in vocabulary learning, whether involving chatbot-assisted language acquisition in L1 or L2, has primarily centered on the question of whether it enhances vocabulary acquisition accuracy, predominantly focusing on the receptive aspect. This limited emphasis is partly attributed to constraints related to research duration and a relative shortage of investigations into long-term memory and productive vocabulary. A more comprehensive examination of these aspects could significantly contribute to the understanding of how to optimize the use of chatbots for promoting vocabulary learning.

### 2.2 Incidental vocabulary learning

Incidental Vocabulary Learning refers to the process of learners unintentionally or non-deliberately acquiring new vocabulary while engaged in specific tasks or activities [15]. This learning method typically is not the learner's primary objective; instead, learners actively pick up these words through exposure to the context in which new vocabulary is encountered. Incidental vocabulary learning is considered a learning approach closest to native language acquisition. Consequently, it has become a prominent focus in second language studies, with recent research emphasizing strategies and tasks conducive to enhancing incidental vocabulary acquisition.

In general, incidental vocabulary learning is thought to occur in everyday life situations. Extensive reading has been the subject of extensive research in this context, and interaction with others has been identified as a significant factor in promoting incidental vocabulary learning [16]. Thus, a personified AI chatbot, by transcending temporal and spatial constraints, has the potential to support learners' second language incidental vocabulary learning through conversation. This research could offer valuable insights and guidance for the application of machine-assisted language teaching.

### 2.3 AI Chatbot in language learning

Chatbots have demonstrated their potential in language learning, particularly in offering definitions, examples, quizzes, and even serving as conversation partners to help students comprehend unfamiliar words and overcome language apprehension [4,[17], [18], [19]]. However, previous research has predominantly focused on rule-based and task-oriented chatbots, which have limitations in providing individualized language instruction. With the growing recognition of individual differences in second language acquisition (SLA), the need for personalized language learning approaches has become apparent [19,20].

In recent years, the emergence of AI has transformed the education landscape, leading to increased research on the application of AI chatbots in linguistics. AI chatbots have revolutionized education by acting as automatic tutors in both virtual and traditional classrooms, offering real-time support and personalized learning experiences. However, existing chatbots have struggled to adapt to the prior knowledge of language learners, and their interactivity has been suboptimal. Students often passively receive information without strong motivation to engage actively. AI chatbots have the potential to address these limitations, particularly in terms of personalized language instruction and increasing learner engagement and motivation [18,21]. Nonetheless, they also face challenges related to technical functionality and concerns about task authenticity [22,23]. Recent technological advancements, particularly the integration of LLMs, hold the promise of resolving these issues [24].

### 2.4 LLMs

**Fig. 1:**
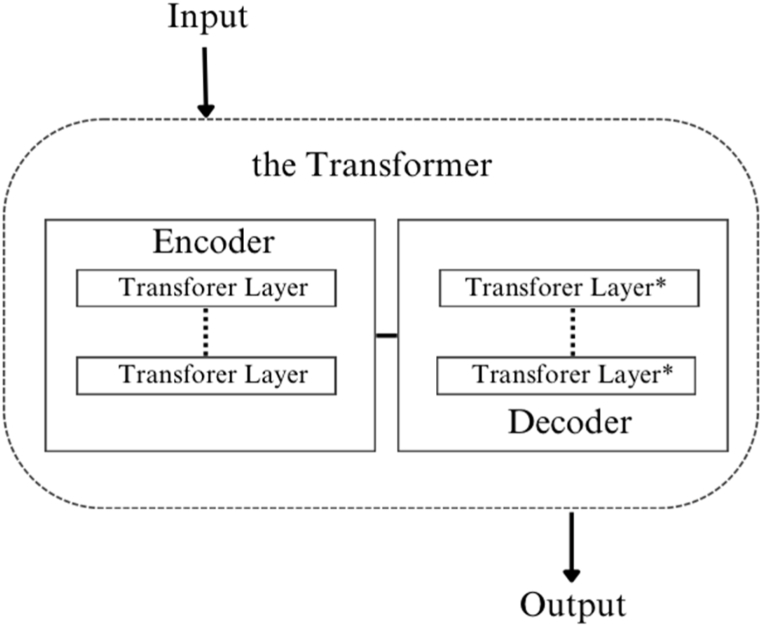
The transformer architecture.

LLMs, such as GPT-3.5 and GPT-4, represent significant breakthroughs in the domain of natural language processing (NLP) [25,26]. At the heart of LLMs lies the Transformer architecture illustrated in Fig. 1, which introduces the self-attention mechanism. This mechanism enables the model to establish connections between different segments of input text, facilitating the handling of lengthy text sequences and the capture of contextual information [27]. It serves as the foundation for LLMs' ability to understand and generate coherent language.

**Fig. 2:**
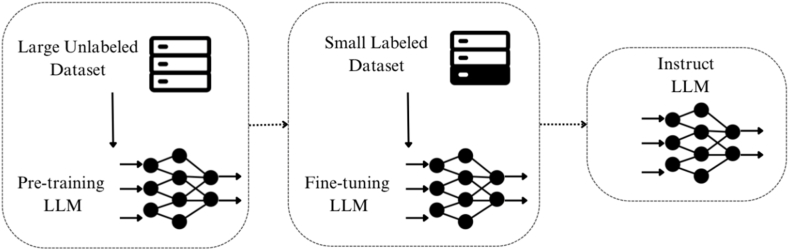
The LLMs for specific purposes.

LLMs are characterized by their vast number of parameters, often reaching into the billions or even hundreds of billions. These parameters are fine-tuned through extensive pre-training on large-scale datasets depicted in Fig. 2 [28]. These datasets are used for pre-training the models, exposing them to a broad spectrum of language patterns and concepts. The diversity and scale of these datasets are instrumental in equipping LLMs with the language understanding and generation capabilities that have garnered their acclaim.

#### 2.4.1 RLHF

**Fig. 3:**
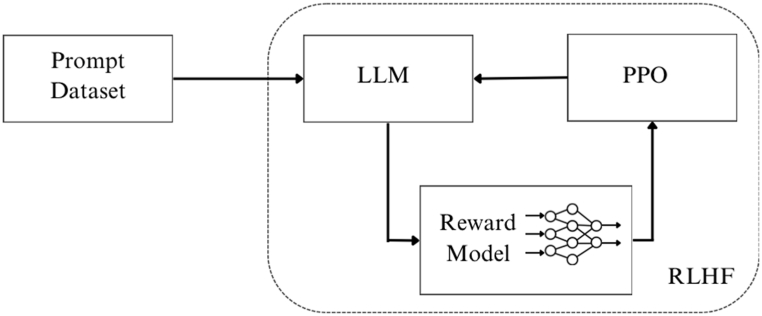
RLHF and proximal policy optimization (PPO).

The innovative training technique that has pushed the boundaries of LLMs is RLHF, which is involved in pre-training as seen in Fig. 3. Daniels-Koch and Freedman state that, RLHF is an attractive replacement for manually specifying goals [29]. The agent in RLHF communicates with its environment, gains or loses points based on its actions, and receives guidance from a human expert. In either case, the human expert can provide either evaluative (such as a rating of the agent's performance) or instructive (such as how to improve) feedback (such as telling the agent what action to take in a particular situation) [30,31].

In traditional Reinforcement Learning (RL), the agent learns solely from the rewards it receives from the environment. In contrast, RLHF introduces an additional layer of feedback from a human expert, enhancing the learning process by offering more informative signals to the agent. Human instructors are presented with two potential trajectories (1, 2) and are asked to choose their favorite. The labels for teachers' preferences are stored in a database, D, represented as a distribution over an interval.

Brian posits that humans are modeled in RLHF techniques as Boltzmann rational decision-makers, controlling the level of decision-making noise through the “rationality” parameter [32]. Equation (1) represents the Boltzmann probability distribution expression, favoring one trajectory over another. In this equation, β controls the level of decision-making noise, which is analogous to the reaction temperature parameter in the Boltzmann model. μ represents the weight parameter corresponding to the chosen trajectory.

Equation (1) is applied to simulate the decision-making process of human experts, comparing trajectory preferences. Equation (2) is employed to assess the accuracy of the model's estimation of the reward function and optimize the model's training. Equation (1) describes the Boltzmann probability distribution expression used to compare the preferences between two trajectories, σ_1_ and σ_2_. Here, P [σ_1_ ≻ σ_2_; β] represents the probability of choosing trajectory σ_1_ over trajectory σ_2_, with β being a parameter that controls decision-making noise. r (σ_1_) and r (σ_2_) respectively denote the actual reward function values for trajectories σ_1_ and σ_2_.

(1) $$P\left[\sigma_{1}≻\sigma_{2};\beta \right]=\frac{\exp\left(\beta \cdot r\left(\sigma_{1}\right)\right)}{\exp\left(\beta \cdot r\left(\sigma_{1}\right)\right)+\exp\left(\beta \cdot r\left(\sigma_{2}\right)\right)}$$

Equation (2) represents the expression for the loss function, which measures the accuracy of estimating the reward function, $\hat{r}$. In this context, loss ($\hat{r}$) is the loss function, and μ(1) and μ(2) are weight parameters associated with trajectories σ_1_and σ_2_, used to balance their respective influences. $\hat{P}$ [σ_1_≻σ_2_] and $\hat{P}$ [σ_2_≻ σ_1_] are the predicted probabilities of trajectory preferences generated by the model, representing the model's estimated trajectory preference distribution.

(2) $$\text{loss}\left(\hat{r}\right)=\mu \left(1\right)\log\hat{P}\left[\sigma_{1}≻\sigma_{2}\right]+\mu \left(2\right)\log\hat{P}\left[\sigma_{2}≻\sigma_{1}\right],\left(\sigma_{1},\sigma_{2},\mu \right)\in D$$

One recent improvement in RLHF involves the implementation of PPO, a policy optimization algorithm in RL introduced by OpenAI in 2017 [33]. It belongs to the class of on-policy algorithms, meaning that it updates the policy using data generated by the current policy. The basic idea behind PPO is to use a clipped surrogate objective function to update the policy. According to Hsu, it is advocated to encourage small-scale policy updates while avoiding large-scale policy changes that may lead to catastrophic failures [34]. This is achieved by introducing a clipping parameter that restricts the alteration in the policy between updates.

In RLHF, PPO can be adopted to learn policies that are optimized for human feedback. This can be achieved by incorporating human feedback into the reward signal of the environment, or by using human feedback as an additional signal to guide policy updates. Recent research has explored various extensions and modifications to the PPO algorithm, including incorporating meta-learning, improving exploration, and addressing the issue of sample inefficiency [33].

### 2.5 Research gap

While the existing research provides valuable insights into LLMs, certain critical areas remain unaddressed. Notably, the impact of LLMs in educational contexts remains relatively limited. These models have the potential to revolutionize education by improving language learning outcomes and facilitating self-directed language learning. Researchers argue that LLMs hold enormous potential to revolutionize education by enhancing teaching, tutoring, and assessment, ultimately elevating the quality of educational materials they provide [24,35]. Additionally, some studies demonstrated substantial improvements in ChatGPT's accuracy, especially in medical education [36]. The application of LLMs in question-and-answer testing in education is an underexplored area.

Researchers have expressed optimism about LLMs in education [24,37], highlighting their potential to enhance learning and teaching experiences, reduce anxiety, increase interest, and provide psychological satisfaction through interactions with AI chatbots. Li et al.'s research stands as a testament to the prowess of LLMs in facilitating Multilingual Learning [38]. Their study delved into the way people perceived the educational benefits when using ChatGPT in various languages discussed within language communities on YouTube. It shows the efficacy of LLMs in addressing the challenges of learning multiple languages, emphasizing their ability to cater to diverse language needs and foster effective language acquisition across various linguistic domains.

Despite the growing interest in LLMs in education, research gaps persist. The impact of AI chatbots, particularly those based on LLMs, on vocabulary learning outcomes and self-directed language learning scenarios is not well understood. There is a need to investigate how LLMs can foster self-directed language learning and improve vocabulary acquisition. This study aims to fill these gaps and provide insights into how LLMs can be optimized for personalized language education.

## 3 Methodology

### 3.1 Research design

**Fig. 4:**
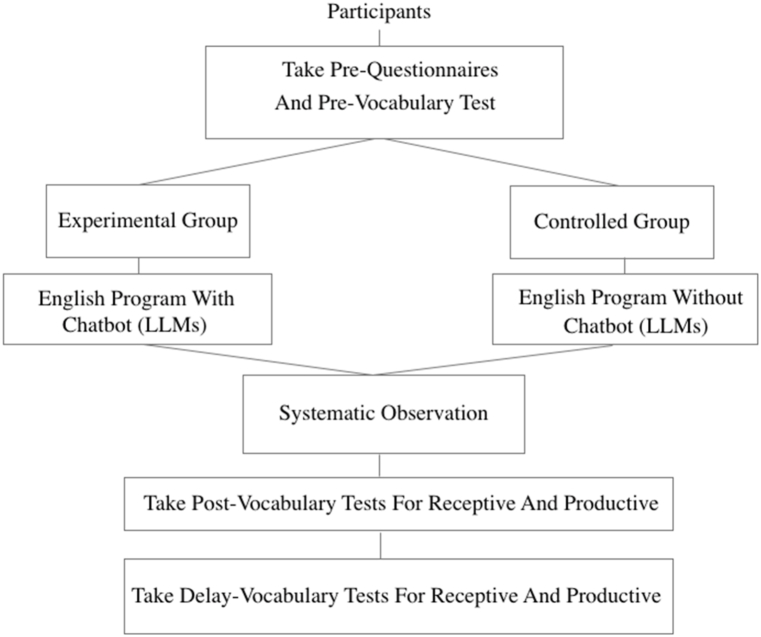
For the research experiment design.

The study adopted a mixed-methods approach, combining both qualitative and quantitative methodologies, to evaluate the impact of Chatbots (LLMs) on vocabulary acquisition among English language learners. The research design followed a quasi-experimental framework with two distinct experimental conditions, both exposed to identical English language learning materials. For a visual representation of the research experiment design, please refer to Fig. 4.

First, participants were randomly assigned to either the experimental or control group. The Experimental Group received access to Chatbots (LLMs) as a learning support tool for vocabulary acquisition, while the Control Group utilized alternative digital resources for vocabulary learning.

Prior to commencing the study, all participants completed an initial vocabulary assessment, ensuring that they exhibited a minimum proficiency level by scoring above the intermediate threshold. The assessment comprised an online version of the Oxford Vocabulary Test with 40 questions (Mean = 44.71 %, Standard Deviation = 0.038). Both groups engaged in identical learning tasks throughout the study, involving the reading of identical texts from the “Cambridge THINK A2” series on a weekly basis. Participants were tasked with identifying the meanings of underlined target words during their designated learning sessions. Progress and learning materials were meticulously controlled to maintain consistency across groups.

The study incorporated systematic observation to monitor and document interactions between the experimental group and the Chatbots (LLMs). Parameters, including interaction frequency, the number of questions, and question types, were recorded. Vocabulary tests were administered bi-weekly to gauge participants' learning progress.

After eight weeks, participants underwent two immediate vocabulary tests: a receptive test and a productive test. The productive test, comprising 18 questions, estimated productive vocabulary knowledge based on the Vocabulary Levels Test by Laufer and Nation [39]. The receptive test, containing 40 questions, was adapted from Read as the Word Associates Test. Data, including accuracy, were collected and analyzed using SPSS [40]. Two post-tests were administered two weeks after the initial tests to assess vocabulary retention.

### 3.2 Participants

A total of 52 participants, comprising 27 females and 25 males, were involved in this study. All participants had a minimum of two years of prior English language study, and their proficiency levels were closely matched. Mandarin Chinese was the participants' first language, and English served as their sole second language. They were high school students who enrolled in an online English course with a primary focus on enhancing their basic interpersonal communication skills (BICS). The course curriculum featured the Cambridge textbook, “THINK,” as its core resource. Apart from this designated textbook, the curriculum ensured consistency in English language learning across both groups by maintaining uniformity in course materials and teaching methods.

Throughout the study, participants completed intermediate-level vocabulary tests, with results disclosed to them, yet no individualized feedback was provided. Comprehensive guidelines were presented to all test subjects, instructing them on how to effectively interact with the AI chatbot for vocabulary acquisition. In the context of this research, both the experimental and control groups engaged in 8-week-long online spring semester courses that commenced at the beginning of the semester. These courses consisted of two weekly sessions, both held in the afternoon.

### 3.3 Chatbot (LLMs) design

#### 3.3.1 System architecture

**Fig. 5:**
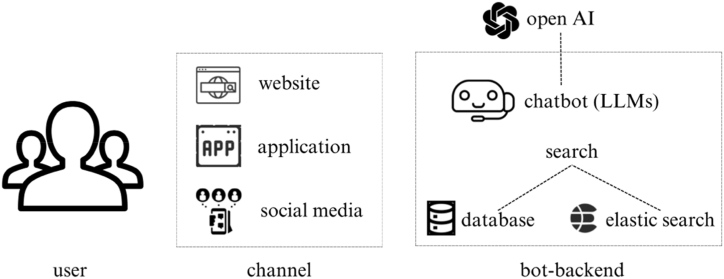
The system architecture of AI chatbot.

This study has developed the system architecture for Chatbots based on LLMs, as depicted in Fig. 5. It consists of three main components: the backend service, search service, and AI robot. These components are integrated into a cohesive system that delivers an effective and reliable chatbot experience for users.

Initially, this study builds the foundation of the Chatbots (LLMs) system architecture, the backend service. This service provides the necessary infrastructure and support to power the chatbot's various features and capabilities. Additionally, it should be scalable, flexible, and easily configurable to meet the business requirements and user needs effectively. Next, this study develops a search service, which enables users to search for information within the chatbot. This service retrieves information from the database and presents it to the user clearly and concisely. It should be optimized for performance to ensure a swift and accurate return of search results. Furthermore, the search service is designed to learn from user interactions and improve search results over time. The most critical component of the chatbot system architecture is the chatbot (LLMs). It is responsible for providing users with intelligent responses to their queries by utilizing natural language processing and machine learning algorithms. To accomplish this, the Chatbots (LLMs) need to be trained on a vast amount of data, including text, audio, and visual content. Also, continuous updates are imperative to remain current with the latest trends and developments in AI technology.

In order to connect these components, this study establishes clear communication channels between them. The backend service communicates with both the search service and the Chatbots (LLMs) to retrieve data and provide it to the user. The search service communicates with the backend service to access the database, and the AI robot communicates with both the search service and the backend service to access information and provide responses.

#### 3.3.2 Learning scenarios and prototype

**Fig. 6:**
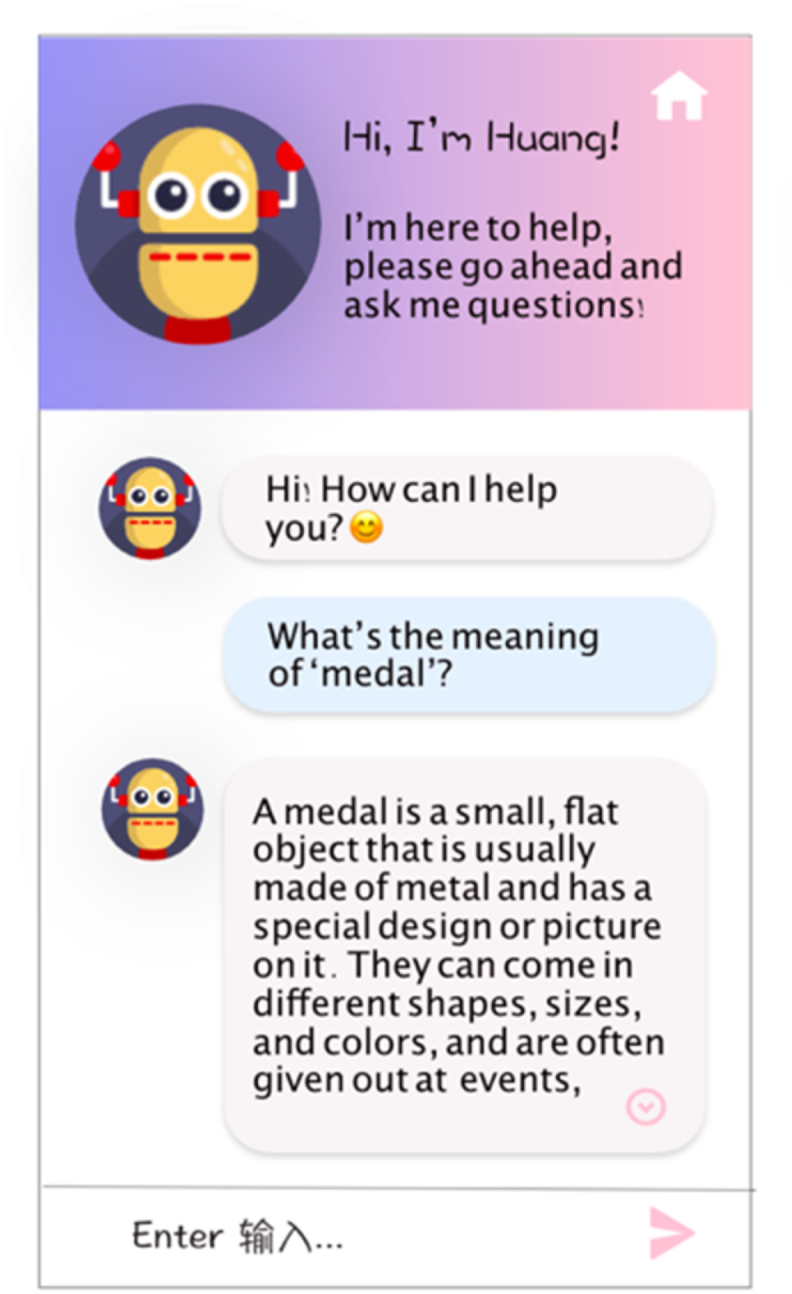
The prototype for chatbots (LLMs).

Chatbots (LLMs) are designed to assist students in their language-learning journey, the prototype of which can be seen in Fig. 6.

Questions support: When a student encounters an unfamiliar word or concept, Chatbots (LLMs) can provide an explanation and examples to aid their understanding. This feature allows students to learn at their own pace and get the necessary support precisely when they need it.

Exercise support: Beyond mere definitions and examples, Chatbots (LLMs) act more like a tutor. They offer a range of teaching activities, such as quizzes and games, to help students practice their language skills and reinforce what they have learned.

Conversation support: Chatbots (LLMs) are trained to respond to conversations about specific topics, such as grammar rules or specific vocabulary words. They can also handle more general conversations, such as greetings and small talk. By engaging with students in this way, Chatbots (LLMs) help them to practice their language skills naturally and immersively.

Personalized Learning: Chatbots (LLMs) can use data from a student's previous interactions to tailor their responses to the student's individual needs. For example, if a student has a low level of English proficiency, Chatbots (LLMs) can provide simpler and more accessible explanations. If a student repeatedly inquires about the meaning of a particular word, Chatbots (LLMs) can provide hints and additional support to aid in remembering the word's meaning.

#### 3.3.3 Learning context

Chatbots (LLMs) are designed to interact with users through natural language processing, which involves understanding human language and generating responses accordingly. Achieving this proficiency requires training chatbots on extensive datasets to discern patterns in the language and respond effectively to user queries. Categorizing chatbot conversations into predefined topics is a useful technique to improve the chatbot's ability to understand and respond to user requests. The conversations between the chatbot and its users can be classified into predefined topics. To understand the user's text and requests, the chatbot must be connected to a particular prompt. Within the Chatbot's (LLM) English-learning system, there are four formal contexts recognized:

1. Vocabulary learning context, which includes the meaning, usage, phrases, examples, synonyms and antonyms, and idioms of words.
2. Conversation context, which focuses on correct grammar, practicing English sentence formation, suggesting expressions, and conducting mock interviews.
3. Text generation context, which involves listing words with the letter “R,” creating stories, writing poems, and suggesting ideas for presentations.
4. Quiz context, which includes multiple-choice, blank-filling, open-ended questions, and corrections.

## 4 Results

### 4.1 Immediate test for receptive and productive vocabulary (EG and CG)

**Fig. 7:**
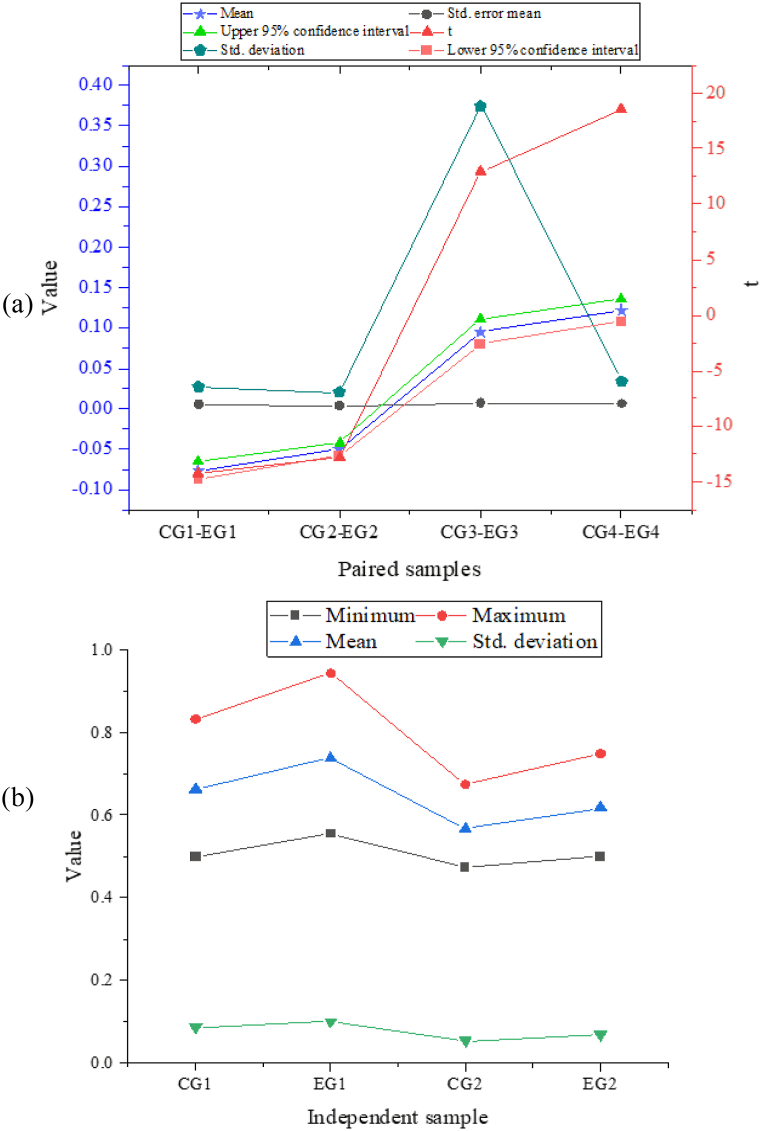
Descriptive statistical results of receptive and productive words ((a) the results of paired sample tests; (b) the independent sample).

Fig. 7 (a) displays the descriptive statistics for means, standard deviation, maximum, and minimum scores of the control group (CG) and the experimental group (EG) in the receptive and productive vocabulary tests. A paired sample *t*-test in Fig. 7 (b) is conducted to examine the differences in performance between the CG and EG in the two types of tests. For the productive test, the mean accuracy score of the CG is 66.24 % (Standard Deviation = 0.086) and that of the EG is 73.93 % (Standard Deviation = 0.078), indicating a statistically significant difference (t = −14.23, p < 0.001). The EG outperforms the CG by 7.6 % on average. Similarly, for the receptive test, the mean accuracy score of the CG is 64.27 % (Standard Deviation = 0.080), and that of the EG is 67.34 % (Standard Deviation = 0.084), indicating a statistically significant difference (t = −12.748, p < 0.001). The EG outperforms the CG by 5.0 % on average. Furthermore, the EG has a higher maximum accuracy score in both tests than the CG. These results suggest that the EG performs significantly better than the CG in both receptive and productive vocabulary tests.

### 4.2 Delay test for receptive and productive vocabulary (EG and CG)

**Fig. 8:**
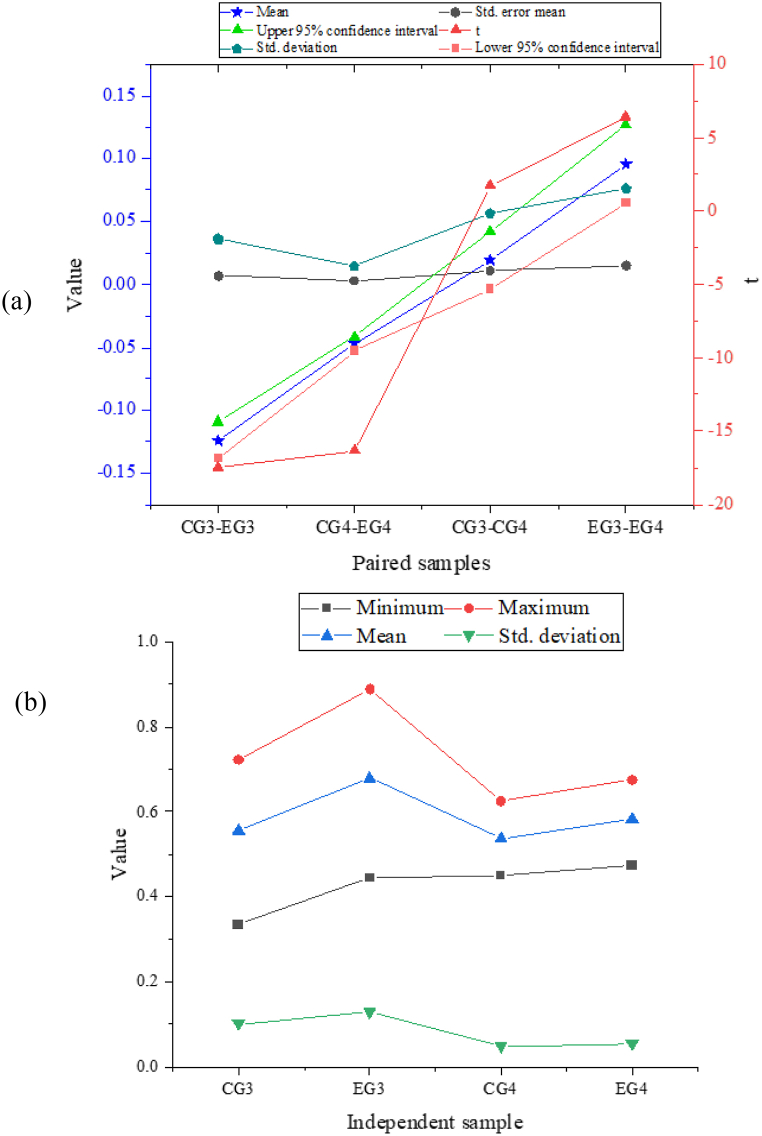
Delay test for receptive and productive vocabulary (EG and CG results) ((a) the test result of paired samples; (b) the independent sample).

The study includes delay tests conducted after a period since the participants are initially exposed to the vocabulary materials. Fig. 8 (a) and (b) presents the descriptive statistics for the control group (CG) and the experimental group (EG) in the delay test. In the productive test, the CG has a mean accuracy score of 55.55 % (Standard Deviation = 0.10), whereas the EG has a mean accuracy score of 67.95 % (Standard Deviation = 0.12), suggesting a significant difference (t = −17.456, p < 0.001). On average, the EG outperforms the CG by 12.39 %. Similarly, in the receptive test, the CG has a mean accuracy score of 53.65 % (Standard Deviation = 0.048), while the EG has a mean accuracy score of 58.37 % (Standard Deviation = 0.084), which shows a significant difference (t = −16.333, p < 0.001). On average, the EG outperforms the CG by 4.7 %. Moreover, the EG achieves a higher maximum accuracy score than the CG in both types of tests. These findings indicate that the EG performs significantly better than the CG in both receptive and productive vocabulary tests, even in the delay test. Furthermore, the advantage of the EG is more pronounced in the productive test when compared to the immediate test.

### 4.3 Systematic observation for EG

This study displays the results of a systematic observation conducted to evaluate the usage and interaction patterns of the Chatbot (LLMs) among students in EG. The observation involves monitoring two primary aspects: the frequency of chatbot usage and the nature of interactions between students and the chatbot, including the number and types of questions asked.

**Fig. 9:**
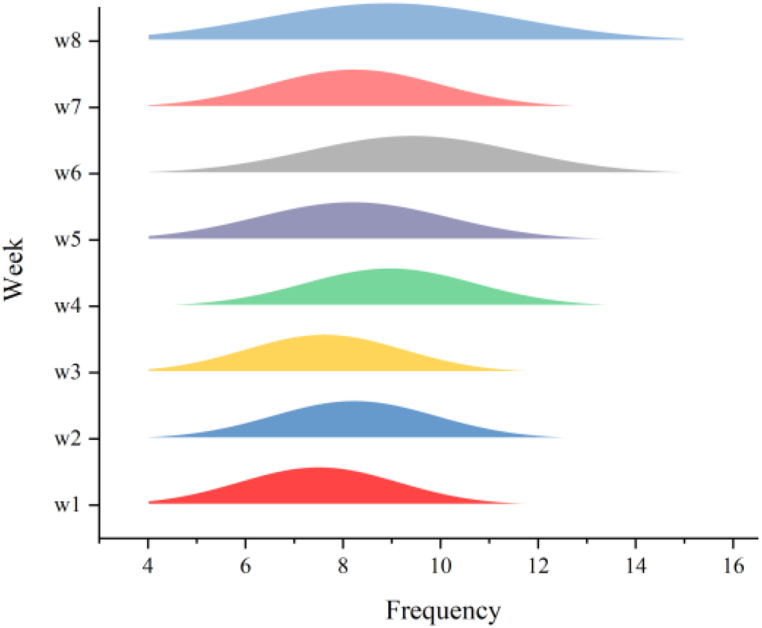
The frequency of using the chatbot (LLMs) in eight weeks.

Fig. 9 presents the frequency of using the Chatbot (LLMs) in a week, which is a crucial indicator for assessing students' engagement with the tool. Students are instructed to initiate a conversation with the chatbot for each word they are assigned to learn. Since word learning tasks are scheduled from Monday to Friday, all students in the EG report using the chatbot more than five times per week. Impressively, 76.92 % of the students indicate that they use the chatbot more than seven times per week. Furthermore, during assessment weeks, the frequency of chatbot usage exhibits an upward trend. Additionally, the interaction frequency between the experimental group and the Chatbot (LLMs) demonstrates an overall increasing trend.

**Fig. 10:**
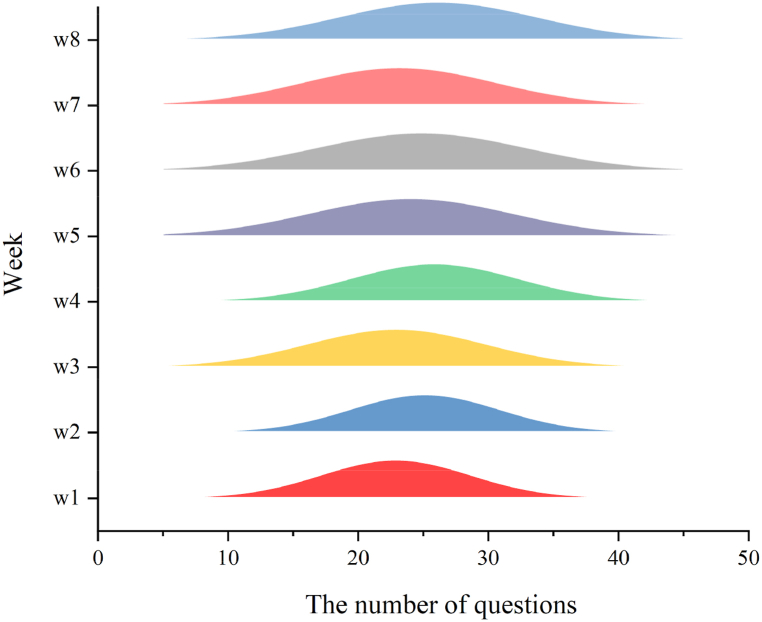
Distribution of questions from eight-week students in chatbot (LLMs).

In order to obtain a more profound insight into these interactions, this study analyzes the number and types of questions posed by students. It's crucial to note that the analysis includes instances where students inquire about both the meaning and example sentences for a given word, counting such interactions as two questions. First, the data presented in Fig. 10 reveal that over 73.07 % of students engage in interactions exceeding 20 questions on average per week. The mean number of questions posed by students weekly stands at 24.37. Similarly, the number of inquiries from the experimental group to the Chatbot (LLMs) shows an overall increasing trend.

**Fig. 11:**
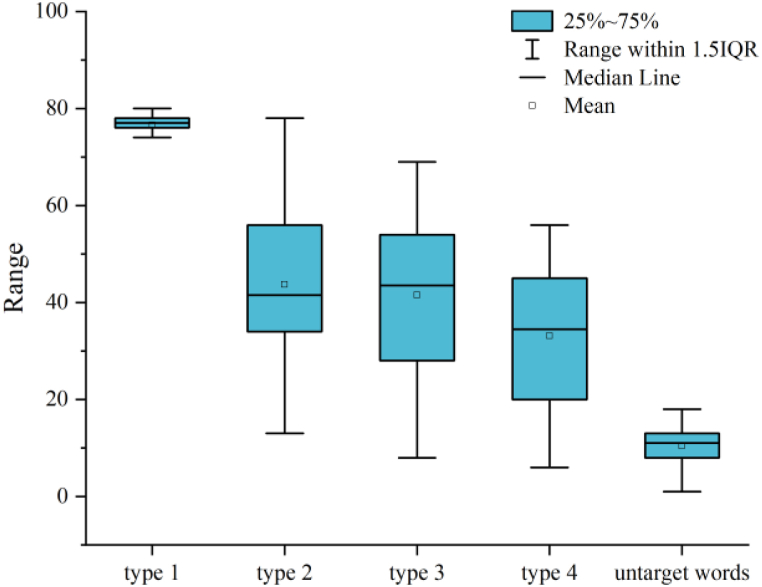
Distribution of question types from eight-week students in chatbot (LLMs).

Second, this study categorizes questions into four types for a more in-depth understanding of student needs, as illustrated in Fig. 11.

The distribution of question types is as follows:

*Type 1 (Meaning Questions):* These questions account for the largest proportion, at 39.29 %. Examples include inquiries about the meaning of a word, such as “What does this word mean?” or “Explain ‘benevolent.'"

*Type 2 (Example Questions):* This category constitutes 22.41 % of the questions. Students often ask how a word is used in a sentence, seeking clarity on usage, such as “How is ‘environment’ used in a sentence?” or “Can you provide some sentences with this word?"

*Type 3 (Antonyms and Synonyms Questions):* Approximately 21.31 % of questions fall into this category. Students inquire about synonyms and antonyms for words and seek related word expressions. This category also included the inquiry for related phrases.

*Type 4 (Contextual Questions):* Questions related to the historical or cultural background of words comprise 16.98 % of the total. These questions are to seek information about the word's context, and its relevance to historical events, cultural traditions, and stories. Students ask, “Is there a historical or cultural context for this word?” or “How is this word used in a given text?” “Could you generate a story with this word?"

Additionally, it is observed that, apart from the designated 80 target vocabulary words, students in the experimental group ask an average of 10.38 questions about non-target words over 8 weeks, with a standard deviation of 4.35.

## 5 Finding and discussion

The primary findings of this research revolve around the effectiveness of AI Chatbots (LLMs) in language learning, specifically focusing on vocabulary acquisition. These findings are in line with prior research, which has demonstrated the positive impact of AI chatbots on second language (L2) vocabulary learning [5,17]. Earlier studies have also explored AI chatbots based on Dialogflow in the context of ESP (Business English) vocabulary learning, concluding that they are valuable tools for engaging students and aiding in the acquisition of ESP English vocabulary [41]. Additionally, some research has delved into the assessment provided by AI chatbots, suggesting that they not only promote vocabulary acquisition but also offer diagnostic insights into individual learners' vocabulary learning progress.

The investigation into the performance of AI Chatbots (LLMs) in vocabulary learning can provide valuable insights to educators, enabling them to develop new pedagogical strategies and techniques for more effective, efficient, and in-depth language learning. This is particularly relevant for the development of productive knowledge, as it can enhance understanding of vocabulary and language skill development.

The first hypothesis aims to examine whether Chatbots (LLMs) enhance receptive vocabulary knowledge in the second language. The analysis of post-test and delay-test results consistently indicates that the experimental group (EG) outperforms the control group (CG) in terms of the accuracy of receptive vocabulary use. Statistical analysis shows that, on average, the EG outperforms the CG by 5.0 % in the post-test and 4.7 % in the delay test.

The second hypothesis focuses on whether Chatbots (LLMs) enhance productive vocabulary knowledge in the second language. Results from both the post-productive and delay tests demonstrate a substantial improvement in the EG compared to the CG. In the post-productive test, the EG scores significantly higher, with an average accuracy of 73.93 % compared to the CG's 66.24 %. The gap between the two groups widens in the delay test, with the EG scoring 67.95 % and the CG scoring 55.55 %. These results suggest that Chatbots (LLMs) are effective in promoting productive vocabulary knowledge, which is traditionally considered more challenging to acquire [13].

The success of Chatbots (LLMs) in enhancing vocabulary acquisition can be attributed to their more engaging interaction style compared to traditional chatbots, which often follow a simple request-response pattern. Research on the dynamics of interactions between second language (L2) learners and near-native speakers, has indicated that engaging with lexical feedback episodes can significantly enhance both productive and receptive language skills [42]. Previous research has primarily focused on motivation and receptive vocabulary learning [18,43], but this study highlights the potential for Chatbots (LLMs) to improve both receptive and productive vocabulary acquisition.

The reasons behind Chatbots (LLMs) promoting receptive vocabulary learning include their alignment with recognized vocabulary learning strategies, such as dictionary use, repetition, and semantic grid strategies [44]. Chatbots (LLMs) provide a platform for knowledge inquiry, offer varied ways of learning the same word, and increase the frequency of target vocabulary words in their responses. Effective strategy utilization influences foreign language proficiency, enhancing specific skills and sub-skills [44].

Regarding the promotion of productive vocabulary learning, one reason is the correlation between enhanced receptive knowledge and increased productive vocabulary. Research [13] indicates that a larger receptive vocabulary size corresponds to a larger productive vocabulary. Furthermore, the experimental group demonstrated a higher level of engagement and posed a greater number of questions, with a significant portion targeting productive knowledge. This heightened involvement of students in language learning is crucial for improving their communicative competence, as good language learners actively guess, communicate, focus on structure and meaning, and monitor their speech and that of others [44].

Chatbots (LLMs) also provide students with opportunities to communicate and reduce language anxiety by offering a safe language output environment. The ability to communicate is essential for enhancing productive vocabulary learning. Chatbots (LLMs) can also address language anxiety by allowing learners to make mistakes in a less judgmental environment, aligning with previous research on the relationship between chatbot usage and learning achievement and technology acceptance [45].

A noteworthy finding is that over 69.23 % of students in the experimental group learned more than 10 non-target vocabulary words, suggesting that Chatbots (LLMs) can promote incidental vocabulary learning in L2 learners. Previous research [45] has shown that task demands, such as attention, retrieval, and generation, lead to higher levels of incidental L2 vocabulary learning. However, the impact of motivation and content on incidental vocabulary learning remains a topic for further research due to the limited sample size and the need for long-term observations.

In conclusion, this study affirms the positive role of AI chatbots, particularly Chatbots (LLMs), in language learning, with a specific emphasis on enhancing productive vocabulary knowledge. Language teachers are encouraged to explore customized AI chatbots tailored to their students' characteristics and learning objectives. As technology continues to evolve, ongoing research is essential to fully harness the potential of AI chatbots in language education and adapt their applications to diverse learning contexts.

## 6 Conclusion

This study has examined the role of Chatbots (LLMs) in enhancing L2 vocabulary learning, employing a robust empirical approach that combined quantitative and qualitative analyses. The findings affirm the positive influence of LLMs on receptive and productive knowledge acquisition, as well as incidental learning, emphasizing their crucial role in enhancing language comprehension abilities.

For language education, this study sheds light on the efficacy of AI chatbots in language learning applications, providing an innovative interactive learning environment. This dynamic interaction with chatbots enriches the understanding of the sociocultural and contextual aspects of language learning, thereby advancing the interactive language learning theory. Furthermore, in the specialized domain of language processing, it has underscored the remarkable performance of LLMs and the transformative nature of the pre-training and fine-tuning paradigm. Chatbots, particularly LLMs, exhibit the capability to generate natural, fluent language responses, contributing to the evolution of natural language generation theory.

The theoretical contributions of this study emphasize the pivotal role of computer-assisted instruction, particularly interactive conversation, in fostering language learning, especially in the domain of vocabulary usage and enhancement. The provision of feedback through chatbot-mediated dialogue is highlighted as a catalyst for productive knowledge. Additionally, this research extends and corroborates the theoretical underpinnings, suggesting a positive impact of AI chatbots on incidental learning in the context of L2 acquisition.

The significance and practical implications of this study are noteworthy. It offers insights into effective teaching strategies for improving productive knowledge in L2 learning by harnessing Chatbots (LLMs) as tutoring aids. Moreover, it advocates the utilization of Chatbots (LLMs) to deliver personalized learning content tailored to individual needs, introducing the concept of personalized learning theory, thereby customizing language learning experiences.

However, acknowledging certain limitations, further research is warranted to delve deeper into the effects of Chatbots (LLMs) on language learning. This includes investigating students' interest and satisfaction levels, changes in motivation, and their impact on incidental learning and self-directed learning. The study should also consider the underlying factors influencing student engagement and participation levels, especially in cases where students do not actively engage with Chatbots (LLMs). Furthermore, future research avenues could explore the potential of Chatbots (LLMs) to enhance cooperation and sharing among learners and their ability to foster higher-order thinking skills, such as reasoning abilities. This continued research will provide a more comprehensive understanding of the educational roles of Chatbots (LLMs) and offer insights for the improvement of chatbots in educational contexts. It is imperative to acknowledge that different types of chatbots may yield varying effects on student learning. Therefore, further research is required to investigate the effects of different chatbot models on language learning comprehensively.

## Ethics statement

All participants/patients (or their proxies/legal guardians) provided informed consent to participate in the study.

## CRediT authorship contribution statement

**Zhihui Zhang:** Writing – review & editing, Writing – original draft, Methodology, Investigation, Formal analysis, Conceptualization. **Xiaomeng Huang:** Software, Formal analysis.

## Declaration of competing interest

The author declare that they have no known competing financial interests or personal relationships that could have appreared to influence the work reported in this paper.
